# Dose estimations for Iranian 11-year-old pediatric phantoms undergoing computed tomography examinations

**DOI:** 10.1093/jrr/rrv017

**Published:** 2015-05-13

**Authors:** Parisa Akhlaghi, Hashem Miri-Hakimabad, Laleh Rafat-Motavalli

**Affiliations:** Physics Department, Faculty of Science, Ferdowsi University of Mashhad, Azadi Square, Mashhad, 91775-1436, Iran

**Keywords:** Iranian 11-year-old phantom, computed tomography, organ depth distribution, dose estimation, Monte Carlo simulation

## Abstract

In order to establish an organ and effective dose database for Iranian children undergoing computed tomography (CT) examinations, in the first step, two Iranian 11-year-old phantoms were constructed from image series obtained from CT and magnetic resonance imaging (MRI). Organ and effective doses for these phantoms were calculated for head, chest, abdomen–pelvis and chest–abdomen–pelvis (CAP) scans at tube voltages of 80, 100 and 120 kVp, and then they were compared with those of the University of Florida (UF) 11-year-old male phantom. Depth distributions of the organs and the mass of the surrounding tissues located in the beam path, which shield the internal organs, were determined for all phantoms. From the results, it was determined that the main organs of the UF phantom receive smaller doses than the two Iranian phantoms, except for the urinary bladder of the Iranian girl phantom. In addition, the relationship between the anatomical differences and the size of the dose delivered was also investigated and the discrepancies between the results were examined and justified.

## INTRODUCTION

Computed tomography (CT) examinations have provided great benefits for patient care, but increased use of CT examinations has raised concerns regarding the enhanced radiation dose and the associated stochastic cancer risk to patients. In addition, compared with adults, pediatric patients are more susceptible to radiation-induced risks owing to their more rapidly growing tissues, their wider and increased cellular distributions of red bone marrow (RBM), and their greater post-exposure life expectancy [1, 2].

Considering that direct measurement of the organ doses is not possible, organ doses are most often calculated by means of Monte Carlo simulations. Computational models of human anatomy are then needed to calculate the dose in the entire body or in specific organs. Heretofore, various pediatric stylized [3] and voxel phantoms [4–9] have been developed, and several authors have reported organ and effective doses for pediatric patients undergoing CT examinations, but these data are valid only for the specific conditions considered in their studies [10]. On the other hand, all the studies focused on dose estimation for pediatric reference models have been based on individuals from Caucasian populations. Furthermore, not all pediatric patients undergoing CT examinations are in 50th percentile of children of their own age-group, and there is great variability between the anatomy of different children [11, 12]. As stated in chapter 21 of handbook of anatomical models for radiation dosimetry because radiation dose depends on the shape and size of the body, estimating the size of the dose for non-reference anatomies is vital. Therefore, non-reference (non-50th percentile) subjects should be modeled to improve patient-specific dose estimates. Such a vast database could provide more accurate estimations of cancer risk and patient-specific organ doses resulting from CT imaging [11].

Currently, a research project is underway to develop a library of Iranian phantoms, which could improve the accuracy of results in studies related to radiological protection and dosimetry in Iran. Considering the importance of modeling non-reference subjects, the purposes of this study were (i) developing two non-reference pediatric 11-year-old phantoms based on CT and MRI images, and (ii) investigating the nature and magnitude of the organ doses resulting from CT examinations of these non-reference phantoms using Monte Carlo simulation. In addition, the resulting organ and effective doses of these two phantoms were compared with those of the University of Florida (UF) 11-year-old male phantom [6, 7] for different examination types and technical settings.

## MATERIALS AND METHODS

### CT scanner simulation

A SOMATOM Sensation 16 multislice CT scanner (Siemens Medical Solutions, Erlangen, Germany) was simulated within the general purpose Monte Carlo radiation transport code, MCNP4C. Scanner characteristics (such as beam angle and focal spot-to-axis distance) and X-ray spectra at different tube potentials were obtained from the manufacturer. The CT scanner has a fan beam angle of 52° and a focal spot-to-axis distance of 57 cm. The scan parameters considered in the simulations are provided in Table 1.

**Table 1.** Scan parameters considered in the simulations

Scan parameters	Tube voltage (kVp)	Collimation (cm)	Pitch	Auto exposure control	Scan coverage			
					Head	Chest	Abdomen–Pelvis	CAP
	80, 100 and 120	1	1	No	From top of the head to the 2nd cervical vertebra	From the clavicles to the middle of the liver	From the top of liver to the midfemoral head	From the clavicles to the midfemoral head

In this research, the same method as that of Khursheed *et al.* was used to define the specific shape of the fan beam [13]. The accuracy of the simulations was verified by comparing the results of the measurements and simulations for CT dose index (CTDI) values. For this purpose, CTDI data were calculated for head and body CTDI phantoms with diameters of 16 and 32 cm, respectively, and were compared with CTDI values measured by Lee *et al.* [10, 14] under the same radiation exposure conditions. Moreover, the peripheral CTDI value at 12 o'clock was measured, and it was then compared with the result of the simulation. A 10-cm pencil-shaped Radcal® ion chamber model 10×5-3CT (Radcal Corporation, Monrovia, CA) and a Radcal 9015 dosimeter (Radcal Corporation, Monrovia, CA) were used to determine the CTDI values [15, 16]. To perform the comparison, the CTDI head and body phantoms were modeled as cylinders having a diameter of 16 cm and 32 cm, respectively, with a length of 15 cm each. The material composition of CTDI phantoms was simulated as polymethylmethacrylate with a density of 1.19 g/cm^3^. The ion chamber was modeled as three 10-cm long concentric cylinders. The innermost cylinder, with a diameter of 0.67 cm, defined the active air volume. The second cylinder, with a diameter of 1.02 cm, defined the chamber wall, which was C552 air-equivalent material with a density of 1.76 g/cm^3^. The third cylinder, with a diameter of 1.37 cm, defined a build-up cap, which was modeled as polyacetal plastic with a density of 1.43 g/cm^3^ [14].

### Pediatric 11-year-old phantoms

Since anatomy and body composition significantly affect the resulting radiation dose, the differences between the dosimetric data for different individuals should be evaluated. Therefore, two Iranian 11-year-old phantoms were developed in Ferdowsi University of Mashhad, using the method described later. Then, these phantoms were used for organ dose estimations and their results were compared with those of the UF voxel 11-year-old male phantom.

#### UF 11-year-old male phantom

The reference voxel phantom of this study was the 11-year-old male phantom of UF Series B, with height of 143.8 cm and weight of 33.59 kg. The UF Series B phantoms were developed from their predecessor UF Series A phantoms, which were in turn constructed through image segmentation of head and CAP CT scans of patients. The UF 11-year-old male phantom was not patient specific, and its anatomical data were closely aligned to those of *ICRP Publication 89* [17]. Due the fact that the age of this phantom did not match with nominal ICRP reference ages, the organ masses and height/weight were interpolated from *ICRP Publication 89* values defined for 10-year-old and 15-year-old children [6, 7].

#### Voxelized model of the Iranian 11-year-old male hybrid phantom

The anatomical model of a male voxel phantom was developed based on image sets of whole-body scan of an Iranian 11-year-old male volunteer. The height and weight of the volunteer were 147 cm and 34.63 kg, respectively.

MRI was used to image the volunteer (instead of CT), based on the ethical considerations (absence of ionizing radiation—especially important for children) and the improved soft tissue contrast of MRI. The volunteer was scanned on a 1.5 T Siemens Magnetom Avanto whole-body scanner at the radiological department of Ghaem Hospital, Mashhad, Iran. The entire scanning time, including breaks for the volunteer, was ∼3 h.

A radiologist, who had expertise in pediatric anatomy, identified the organs and tissues in the MRI images. Based on his identification, manual segmentation was performed using 3D-DOCTOR^TM^ (Able Software Corp., Lexington, MA), a 3D modeling and image-processing software package. The sagittal, axial and coronal images were imported into 3D-DOCTOR, and the anatomical structures of interest were contoured manually using a computer mouse. About 104 different tissues and organs were identified and segmented for the model.

Polygon mesh models were rendered from the resulting segmented images. All the organ mesh models were imported to Rhinoceros (McNeel, Seattle, WA, USA), a NURBS modeling tool. Using Rhinoceros, they were oriented and their locations were adjusted. Due to the defects of some mesh surfaces, NURBS surfaces were developed for some organs and tissues, to improve their models. All of these mesh and NURBS models form an anatomical realistic boundary representation (BREP) phantom. To incorporate the geometries into Monte Carlo code, all organs of the model were voxelized using a voxelizer developed by our research group. This in-house voxelizer was written in FORTRAN code, with the same method used by Lee *et al.* at the University of Florida [11]. The resulting voxel resolution was 0.15 cm × 0.15 cm × 0.3 cm, and the voxel array size was 300 × 170 × 490. Finally, the density and the elemental composition of organs and tissues of UF pediatric phantoms were attributed to those of the Iranian phantom. Further information about phantom construction can be found in our previous paper [18].

#### Voxelized model of the Iranian 11-year-old female hybrid phantom

The Iranian 11-year-old female phantom in this study was constructed from CAP CT images of a pediatric patient examined at Imam Hossein Hospital in Tehran, Iran. Because only the images of the CAP scan were available, a mesh model of the whole body, which was comparable with the trunk model obtained from CT images, was created using MakeHuman, an open source 3D computer graphics application [19]. In this process, some parameters like body diameters, trunk height, and shoulder width of the default 11-year-old child defined in MakeHuman were changed, so that its trunk was matched to the trunk boundary obtained from the CT images. By changing these parameters, MakeHuman modifies the sizes of other body parts in proportion to the size of the phantom trunk. Because MakeHuman only provides the size, shape and body boundary of the phantom, and it does not contain internal organs, polygon mesh models for the brain, legs and arms were obtained from those of the Iranian 11-year-old male phantom. These models were rescaled, so that they were matched with the body boundaries of the Iranian girl. Finally, the same method described above was used to construct the Iranian female phantom using mesh and NURBS models of the organs. The resulting voxel resolution was 0.15 cm × 0.15 cm × 0.3 cm, and the voxel array size was 250 × 150 × 480.

### Organ and effective dose estimations

In this study, organ doses from axial scans were approximated for the head, chest, abdomen–pelvis and CAP examinations. A total of 35 organs and tissues, including RBM, were involved in the organ dose calculations under tube potentials of 80, 100 and 120 kVp. Assuming charged particle equilibrium (CPE), the absorbed dose was approximated by collision kerma and was recorded using F6:p tally, which estimates energy deposition averaged over a cell. The simulations provide the dose in MeV/g, i.e. energy deposition (MeV) per unit mass (g). Considering that the unit of absorbed dose is Gy (J/kg), the outputs of the program should be multiplied by 1.6 × 10^−10^. It is worth mentioning that three different parts of bones (spongiosa, cortical bone and medullary cavity) of Iranian phantoms were defined explicitly in the voxel models, so their RBM absorbed doses could be determined based on the method recommended in *ICRP Publication 116* [20], whereas in the UF voxel phantom, different parts of the bones were not distinguished. As a consequence, in order to have comparable results, a similar method was used to calculate the absorbed doses of the RBM for all phantoms, so a homogeneous mixture was considered for bones of Iranian and UF phantoms. For calculation of the RBM absorbed dose, the F4 tally was used. This tally provides the volume flux in cubic centimeters. Therefore, the outcomes of this tally were multiplied by the flux to absorbed dose coefficients (mGy.cm^3^), to obtain the RBM dose in mGy.

The same scan parameters and anatomical landmarks were used for each type of CT examination of both UF and Iranian phantoms. A total of 10^9^ photons were simulated to obtain reasonable relative errors of <2% for major organs and tissues located in the scan coverage. Errors were obviously higher for the tissues located outside of the scan region.

Effective doses for a range of phantoms and tube potentials were also included in the calculations. Tissue-weighting factors introduced in *ICRP Publication 103* [21] were used to compute effective dose from the equivalent doses for different organs and tissues.

### Calculation of depth distributions of main organs

For a more detailed study of organ dose discrepancies between phantoms, anatomical differences in the scan field should be reviewed. In addition to the attenuation properties of overlying tissues and organs, the depth below the surface is also a parameter that significantly influences the dose from external radiation. Depth of organs from the body surface indicates the extent of the overlying tissues, by which each point of an organ or tissue is shielded from radiation impinging from X-ray source rotating around the body.

Thus, depth distribution below the body surface was calculated for the main organs of the 11-year-old phantoms, and its effect on the amount of dose was investigated. The distributions were evaluated for 1 million points sampled randomly in the organs under consideration [22]. For this purpose, the average distance of each of 1 million points from the skin voxels located in the same slice of that point was calculated. Using this data in the calculations, the distance between each voxel of each organ and all the skin voxels existing around the body was specified, these distributions were valid for a full rotation of the X-ray source. In addition to depth distributions, the amounts of soft tissue, adipose tissue and bone were determined in each scan region to investigate the extent of the overlying tissues that shield the internal organs.

## RESULTS

### Model validation

Four different point doses (the central dose and the doses at 12, 3 and 6 o'clock positions) were determined within the CTDI head and body phantoms using the ion chamber at a collimation of 1 cm under three tube potentials of 80, 100 and 120 kVp. The weighted CTDI (CTDI_w_), which is defined as the summation of one-third of CTDI_center_ and two-thirds of CTDI_periphery_, was 6.20, 11.60 and 16.20 mGy, for the CTDI head phantom at tube voltages of 80, 100 and 120 kVp, respectively. The maximum error between the results of the simulation and measurements was almost 9% for all tube potentials [10, 14].

In addition, it was observed that there is a good agreement between the results of peripheral CTDI values obtained by simulation and measurement (<5% difference). For instance, the measured and simulated values of peripheral CTDI at 12:00 at a tube voltage of 80 kVp were 6.83 and 6.53 mGy, respectively [15, 16].

### The Iranian 11-year-old pediatric phantoms

**Fig. 1.**
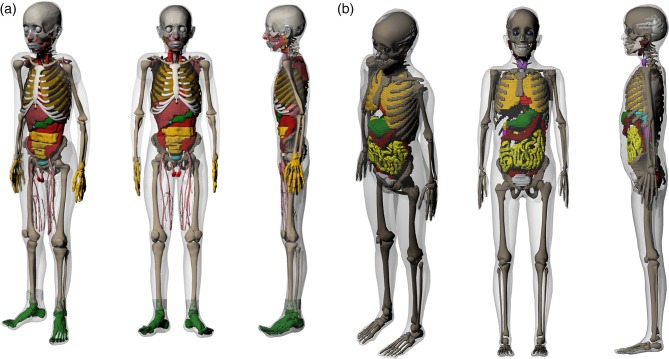
Iranian hybrid phantoms: (**a**) 11-year old male phantom, and (**b**) 11-year old female phantom.

**Fig. 2.**
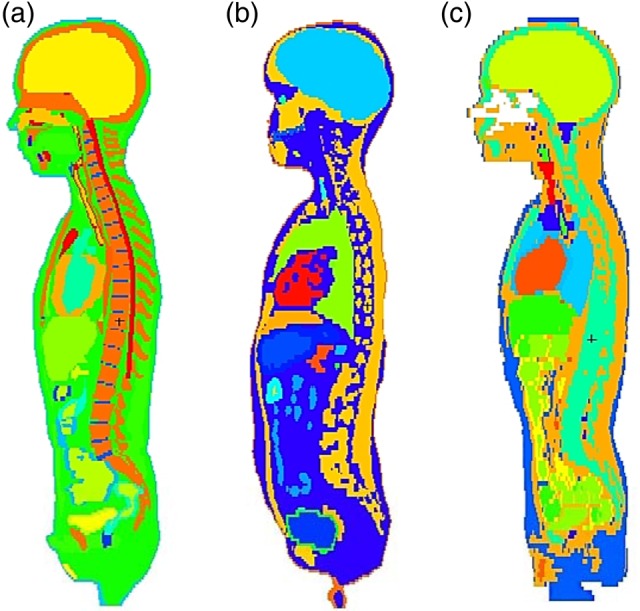
The sagittal views of pediatric phantoms: (**a**) Iranian 11-year-old male phantom, (**b**) Iranian 11-year-old female phantom and (**c**) UF 11-year old male phantom.

**Fig. 3.**
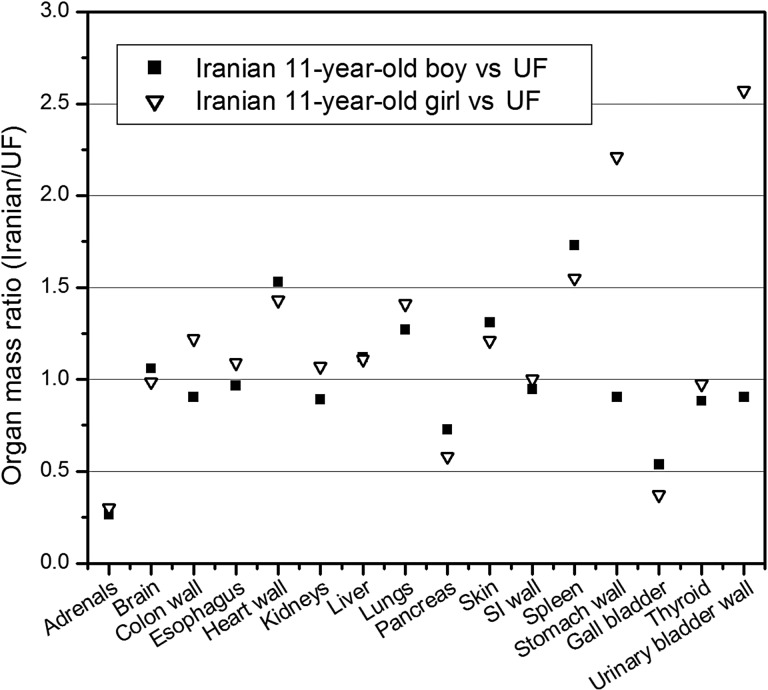
Organ mass ratio of Iranian and UF 11-year-old phantoms.

Figure 1 represents the resulting hybrid models of the Iranian 11-year-old male (Fig. 1a) and Iranian 11-year-old female (Fig. 1b). Moreover, the sagittal views of the Iranian and UF 11-year-old phantoms are displayed in Fig. 2. Table 2 contains the masses of some organs, total mass, height and body mass index (BMI) of these phantoms. It should be noted that these two developed phantoms are not Iranian standard phantoms. In addition, Fig. 3 displays the organs mass ratio of Iranian non-reference and UF reference phantoms.

**Table 2.** The masses of some organs, total mass, height and BMI of Iranian and UF 11-year-old phantoms

Organs	Mass (kg)		
	UF	Iranian boy	Iranian girl
Adrenals	7.61E-03	2.02E-03	2.30E-03
Brain	1.42E+00	1.50E+00	1.40E+00
Colon wall	2.28E-01	2.06E-01	2.78E-01
Esophagus	2.04E-02	1.97E-02	2.23E-02
Heart wall	1.59E-01	2.44E-01	2.28E-01
Kidneys	1.94E-01	1.73E-01	2.07E-01
Liver	8.40E-01	9.43E-01	9.29E-01
Lungs	5.80E-01	7.35E-01	8.19E-01
Pancreas	7.16E-02	5.21E-02	4.15E-01
Skin	1.40E+00	1.83E+00	1.69E+00
Small intestine wall	4.00E-01	3.79E-01	4.00E-01
Spleen	9.14E-02	1.58E-01	1.42E-01
Stomach wall	9.12E-02	8.24E-02	1.61E-01
Gall bladder	1.76E-02	9.47E-03	6.54E-03
Gonads	4.84E-03	5.59E-03	2.57E-03
Thyroid	8.70E-03	7.68E-03	8.48E-03
Urinary bladder wall	2.85E-02	2.58E-02	5.53E-02
Total mass (kg)	33.59	34.63	31.33
Height (cm)	143.8	147	142.2
BMI (kg.m^−2^)	16.24	16.02	15.49

Fifty percent of organs and tissues in the Iranian boy phantom and the UF phantoms show relative differences of >20% in their masses. Among those organs, the adrenals and spleen have the maximum relative differences (73.5% and 72.9%, respectively). The masses of all organs of the Iranian girl agree with those of the UF phantom within 50%, except for the adrenals, spleen, gall bladder, stomach and urinary bladder.

The large discrepancies are mainly observed for organs in the gastrointestinal and urinary tracts (small intestine, bladder and esophagus), which are highly variable even in a single person according to amount of time passed since having a meal. It should be noted that for the wall of organs (e.g. the colon of the two Iranian phantoms), the same quantity of voxels were attributed.

There are also significant differences between the mass of the gall bladder in these phantoms. Between meals, bile secreted from the liver is stored in the gall bladder. During a meal, bile is injected from the gall bladder into the small intestine. Consequently, gall bladder shows considerable variations in shape and size. In addition, there are considerable discrepancies between the masses of lungs and hearts for these phantoms due to their continuous moving during MRI scanning.

### Dose estimations

**Fig. 4.**
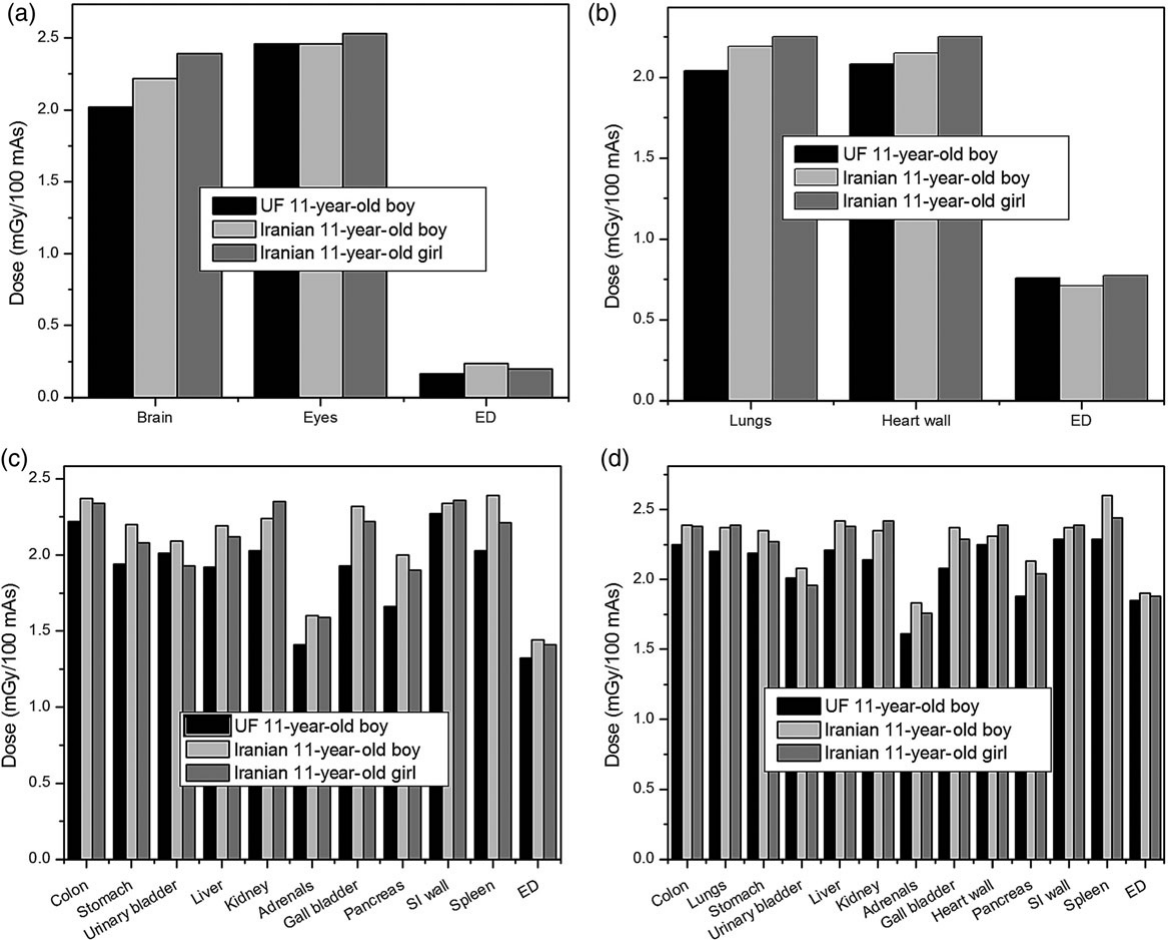
Comparisons between absorbed doses of main organs and effective doses of Iranian and UF phantoms for (**a**) head, (**b**) chest, (**c**) abdomen–pelvis, and (**d**) CAP scan at a tube voltage of 80 kVp.

Organ and effective doses determined for the UF and Iranian 11-year-old phantoms, together with their statistical errors, at tube potential of 120 kVp are tabulated in Table 3 for head, chest, abdomen–pelvis, and CAP scans. In addition, the comparisons between the absorbed doses of organs exposed directly in the scan region for these phantoms at a tube voltage of 80 kVp are plotted in Fig. 4.

**Table 3.** Absorbed doses for organs (in mGy/100 mAs) and effective doses (in mSv/100 mAs) of 11-year-old phantoms, together with their absolute statistical errors for varying scan coverage at a tube voltage of 120 kVp

	Head scan			Chest scan			Abdomen–pelvis scan			CAP scan		
	UF	Iranian boy	Iranian girl	UF	Iranian boy	Iranian girl	UF	Iranian boy	Iranian girl	UF	Iranian boy	Iranian girl
**Colon**	0.01600 ± 0.00007	0.01900 ± 0.00011	0.02100 ± 0.00011	0.27700 ± 0.00028	0.28400 ± 0.00043	0.60000 ± 0.00054	8.30000 ± 0.00249	8.73000 ± 0.00262	8.57000 ± 0.00257	8.42000 ± 0.00253	8.84000 ± 0.00354	8.76000 ± 0.00350
**Lungs**	0.17400 ± 0.00023	0.15100 ± 0.00021	0.14300 ± 0.00020	7.57000 ± 0.00151	7.98000 ± 0.00160	8.17000 ± 0.00163	2.12000 ± 0.00106	2.60000 ± 0.00104	2.53000 ± 0.00101	8.23000 ± 0.00247	8.70000 ± 0.00261	8.76000 ± 0.00263
**Stomach**	0.04900 ± 0.00022	0.03400 ± 0.00021	0.04000 ± 0.00019	3.03000 ± 0.00182	1.99000 ± 0.00159	3.40000 ± 0.00170	7.22000 ± 0.00361	8.07000 ± 0.00404	7.69000 ± 0.00308	8.22000 ± 0.00493	8.72000 ± 0.00523	8.47000 ± 0.00424
**Gonads**	0.00900 ± 0.00045	0.01300 ± 0.00041	0.00900 ± 0.00043	0.01600 ± 0.00042	0.02300 ± 0.00052	0.03600 ± 0.00082	5.80000 ± 0.01044	6.89000 ± 0.01102	6.75000 ± 0.01485	5.83000 ± 0.01224	5.26000 ± 0.01157	6.82000 ± 0.01705
**Urinary bladder**	0.00008 ± 0.00015	0.01300 ± 0.00022	0.01000 ± 0.00012	0.02000 ± 0.00022	0.03500 ± 0.00033	0.02800 ± 0.00019	7.75000 ± 0.00620	7.88000 ± 0.00709	7.22000 ± 0.00433	7.76000 ± 0.00776	7.85000 ± 0.00785	7.34000 ± 0.00514
**Liver**	0.05600 ± 0.00017	0.04100 ± 0.00014	0.04700 ± 0.00015	3.94000 ± 0.00118	3.30000 ± 0.00132	4.61000 ± 0.00138	7.18000 ± 0.00287	8.00000 ± 0.00240	7.79000 ± 0.00234	8.34000 ± 0.00334	8.92000 ± 0.00357	8.80000 ± 0.00352
**Esophagus**	0.31200 ± 0.00094	0.60700 ± 0.00127	1.52000 ± 0.00198	5.31000 ± 0.00372	5.69000 ± 0.00398	4.64000 ± 0.00325	2.29000 ± 0.00366	1.32000 ± 0.00277	1.77000 ± 0.00283	6.33000 ± 0.00696	6.18000 ± 0.00680	5.16000 ± 0.00568
**Thyroid**	0.81100 ± 0.00292	1.40000 ± 0.00392	1.25000 ± 0.00363	6.93000 ± 0.00762	2.62000 ± 0.00498	1.91000 ± 0.00420	0.33600 ± 0.00265	0.21700 ± 0.00208	0.23500 ± 0.00212	7.08000 ± 0.01274	2.74000 ± 0.00822	2.01000 ± 0.00704
**Brain**	7.70000 ± 0.00154	8.20000 ± 0.00164	8.63000 ± 0.00173	0.12000 ± 0.00019	0.09200 ± 0.00017	0.09600 ± 0.00018	0.03100 ± 0.00015	0.03200 ± 0.00014	0.03600 ± 0.00016	0.14300 ± 0.00037	0.11600 ± 0.00032	0.12200 ± 0.00034
**Adrenals**	0.03800 ± 0.00052	0.03500 ± 0.00087	0.03200 ± 0.00072	2.05000 ± 0.00349	2.44000 ± 0.00659	2.09000 ± 0.00543	5.77000 ± 0.00866	6.37000 ± 0.01465	6.38000 ± 0.01340	6.63000 ± 0.01061	7.31000 ± 0.01828	7.07000 ± 0.01626
**Gall bladder**	0.03800 ± 0.00055	0.02400 ± 0.00047	0.02800 ± 0.00062	1.55000 ± 0.00341	0.63700 ± 0.00242	1.23000 ± 0.00369	7.16000 ± 0.01074	8.56000 ± 0.01113	8.22000 ± 0.01233	7.80000 ± 0.01248	8.81000 ± 0.01322	8.58000 ± 0.01544
**Heart**	0.13800 ± 0.00036	0.11100 ± 0.00033	0.11200 ± 0.00038	7.68000 ± 0.00230	7.93000 ± 0.00238	8.24000 ± 0.00330	2.33000 ± 0.00186	2.27000 ± 0.00182	2.13000 ± 0.00213	8.40000 ± 0.00420	8.60000 ± 0.00430	8.83000 ± 0.00530
**Kidneys**	0.03100 ± 0.00019	0.02900 ± 0.00020	0.02600 ± 0.00017	1.17000 ± 0.00105	1.19000 ± 0.00119	0.92900 ± 0.00102	7.68000 ± 0.00384	8.34000 ± 0.00417	8.65000 ± 0.00433	8.18000 ± 0.00491	8.81000 ± 0.00529	8.95000 ± 0.00448
**Pancreas**	0.03100 ± 0.00026	0.02900 ± 0.00030	0.03000 ± 0.00032	1.34000 ± 0.00161	1.43000 ± 0.00200	1.82000 ± 0.00237	6.93000 ± 0.00554	7.65000 ± 0.00612	7.37000 ± 0.00590	7.51000 ± 0.00676	8.21000 ± 0.00739	7.95000 ± 0.00795
**Small intestine**	0.01600 ± 0.00008	0.01800 ± 0.00011	0.01800 ± 0.00010	0.28300 ± 0.00034	0.20400 ± 0.00037	0.27500 ± 0.00036	8.49000 ± 0.00255	8.67000 ± 0.00347	8.68000 ± 0.00260	8.62000 ± 0.00345	8.77000 ± 0.00351	8.79000 ± 0.00352
**Spleen**	0.05300 ± 0.00034	0.04002 ± 0.00027	0.04800 ± 0.00029	3.54000 ± 0.00248	2.69000 ± 0.00188	4.76000 ± 0.00286	7.51000 ± 0.00526	8.60000 ± 0.00516	8.02000 ± 0.00481	8.55000 ± 0.00684	9.42000 ± 0.00565	8.97000 ± 0.00628
**Prostate/** **Uterus**	0.00700 ± 0.00052	0.00900 ± 0.00037	0.00800 ± 0.00019	0.01500 ± 0.00055	0.02400 ± 0.00061	0.02500 ± 0.00033	6.65000 ± 0.01663	6.59000 ± 0.01318	6.06000 ± 0.00667	6.64000 ± 0.01992	6.49000 ± 0.01558	6.14000 ± 0.00798
**Eyes**	8.66000 ± 0.02165	8.64000 ± 0.02506	8.76000 ± 0.03154	0.13200 ± 0.00260	0.11400 ± 0.00307	0.11300 ± 0.00364	0.04700 ± 0.00231	0.05400 ± 0.00308	0.05100 ± 0.00334	0.16000 ± 0.00486	0.15700 ± 0.00595	0.14600 ± 0.00657
**Bone**	3.80000 ± 0.00076	2.99000 ± 0.00060	3.13000 ± 0.00063	3.97000 ± 0.00040	3.67000 ± 0.00073	4.45000 ± 0.00089	7.17000 ± 0.00143	6.49000 ± 0.00130	7.55000 ± 0.00151	10.40000 ± 0.00208	9.38000 ± 0.00188	11.10000 ± 0.00222
**RBM**	1.83000 ± 0.00064	2.52000 ± 0.00088	1.83000 ± 0.00064	1.56000 ± 0.00117	2.39000 ± 0.00179	2.14000 ± 0.00161	2.79000 ± 0.00488	3.10000 ± 0.00543	3.02000 ± 0.00559	4.06000 ± 0.00426	5.08000 ± 0.00533	4.71000 ± 0.00495
**Effective dose**	0.61200 ± 0.00440	0.87600 ± 0.00617	0.73500 ± 0.00573	2.86000 ± 0.01027	2.67000 ± 0.00894	2.88000 ± 0.00867	4.96000 ± 0.00694	5.32000 ± 0.00867	5.32000 ± 0.00793	6.96000 ± 0.00631	7.06000 ± 0.00749	7.07000 ± 0.00742

From Table 3 and Fig. 4, it is observed that all organs of the Iranian male phantom exposed directly in the scan range receive more doses than those of the UF phantom. The same trend is observed for the Iranian female phantom, except for its urinary bladder.

The relative differences between the UF and Iranian male absorbed doses of the brain (in the head scan), lungs (in the chest scan) and colon (in the abdomen–pelvis scan) are almost 10%, 7.3% and 6.7%, separately, at a tube voltage of 80 kVp. The relative differences between the UF and Iranian female absorbed doses of the brain (in the head scan), lungs (in the chest scan), colon (in the abdomen–pelvis scan), are almost 18.3%, 10.3% and 5.4%, respectively. Increasing the tube voltage decreases the relative differences between the organs doses of the UF and Iranian phantoms.

### Depth distributions of the main organs and tissues

**Fig. 5.**
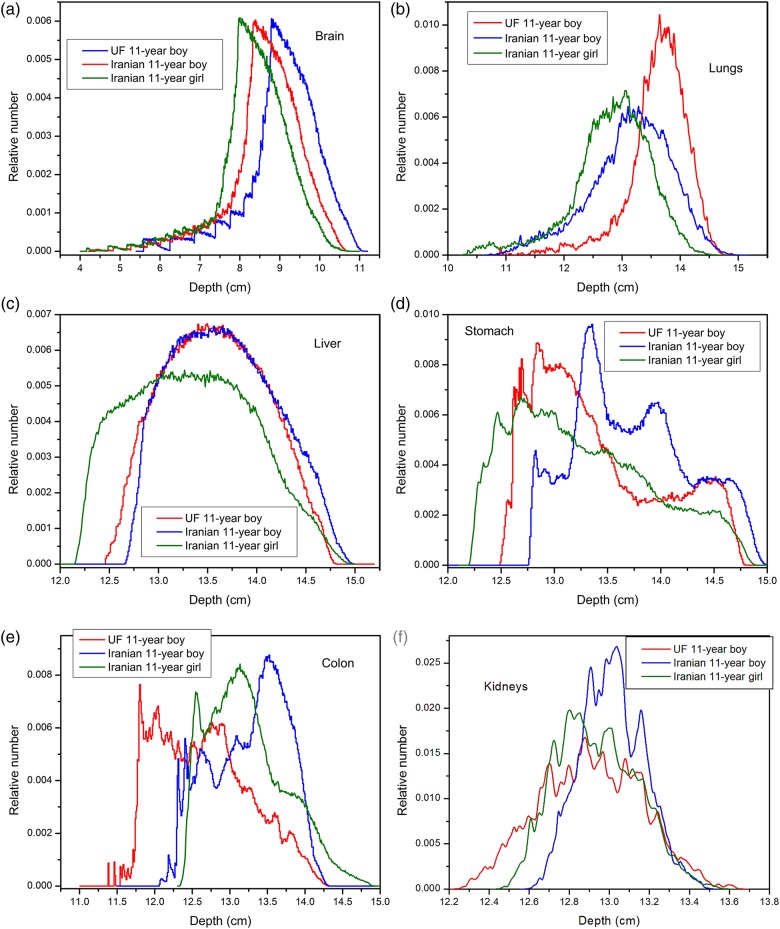
Depth distributions of brain, lungs, liver, stomach, colon and kidneys of Iranian and UF 11-year-old phantoms.

The depth distributions of some of the main organs located in the scan range are displayed in Fig. 5. In this figure, depth distributions of the brain in the head scan, the lungs in the chest scan, and the liver, stomach, colon and kidneys in abdomen–pelvis scan are plotted. According to the figure, compared to the UF reference phantom, the Iranian phantom organs located in the head and chest regions are closer to the body surface. In the abdomen–pelvis region, compared with other phantoms, all main organs of the Iranian male are seated deeper in the body.

### Quantities of overlying tissues

**Fig. 6.**
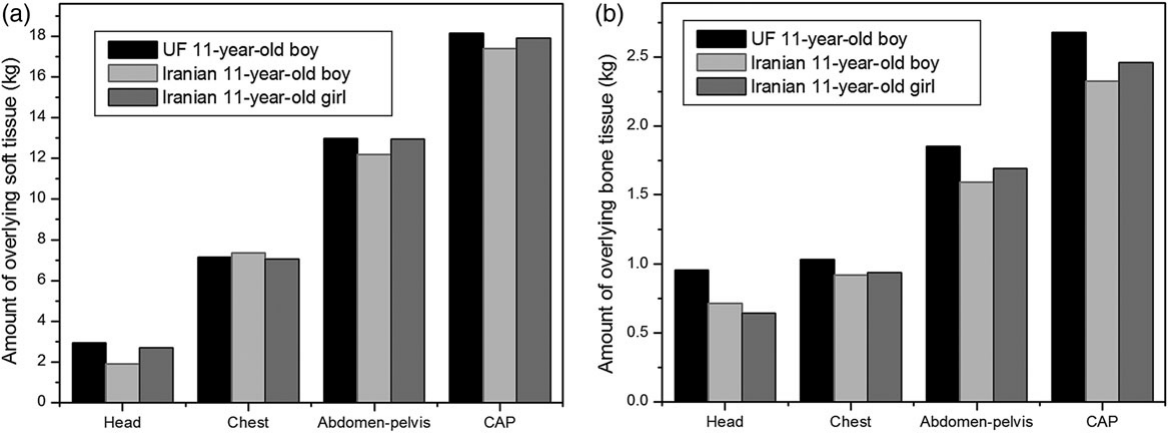
Amount of overlying soft (adipose and muscle) tissue and bone tissue of Iranian and UF phantoms for head, chest, abdomen–pelvis and CAP scans.

Figure 6 displays the quantities of overlying bones and tissues (adipose and muscle) in each scan range for these phantoms. Depending on the quantities of soft tissue, adipose and bone for these phantoms in chest and head scans, all the main organs benefit from almost the same level of protection from the external exposure, while in the abdomen–pelvis and CAP scans, all organs of the UF phantom, which are exposed directly, are shielded more than those of the Iranian phantoms are.

## DISCUSSION

In order to further validate the results, the absorbed doses calculated in this study for the UF reference phantom were compared with those reported by Lee *et al.* for the 10-year-old hybrid phantom. Using the same method as Lee *et al*., organ doses from our study were normalized to the CTDI data obtained in our calculations to eliminate the effects of the CT scanner–specific features, such as inherent X-ray tube output [10]. Considering the value of CTDI_vol_ reported on our Siemens Sensation 16 scanner, it was observed that the relative differences between the absorbed doses of the main organs of the 10-year-old hybrid phantom and the UF 11-year-old phantom are <13%. For instance, the absorbed doses for the brain (in the head scan), lungs (in the chest scan) and colon (in the abdomen–pelvis scan) of the 10-year-old hybrid at a tube voltage of 120 kVp are 12.50, 12.50 and 13.80 mGy/100 mAs, respectively; these values for the UF 11-year-old phantom are 11.07, 10.89 and 11.94 mGy/100 mAs, respectively. Given that these phantoms are not for the same age, these differences seem reasonable.

Given the results of dose estimations, some discrepancies were observed between the absorbed doses to the organs for these three phantoms. The results of dose estimations at 80 kVp showed that the maximum relative difference between the doses of the UF and the two Iranian phantoms (20% for the gall bladder) is more than the maximum relative difference between the two Iranian phantoms (8% for the urinary bladder). To find out the reasons of this behavior, the anatomical discrepancies between these 11-year-old phantoms were studied.

Considering the depth distributions of the brain and left lung (Fig. 5), and due to the fact that in each of these phantoms, organs located in the chest and head regions are shielded almost equally, receiving the maximum doses to the brain (in the head scan), heart wall and lungs (in the chest scan) by Iranian girl is justified. In this regard, it could be said that the greater depths and greater amounts of overlying tissues for the UF internal organs cause the minimum dose to be absorbed by the brain (in the head scan), and by the heart wall and lungs (in the chest scan).

In the abdomen–pelvis and CAP scans, the main organs of the Iranian phantoms always receive greater doses than those of the UF phantoms, except for the urinary bladder of the Iranian girl phantom, which could be due to the greater volume of the Iranian girl urinary bladder compared with those of other phantoms. Besides, given the organs depth distributions of these three phantoms (Fig. 5) in abdomen-pelvis and CAP scans, it is expected that organs of UF phantom receive more amounts of dose. This remark is in contrast with the results of simulations, in which UF organs absorb less amounts of dose. To explain this issue, we need to consider BMI, which is a measure of relative size based on the mass and height of an individual, and which is representative of body fatness, and independent of age, sex and ethnicity [23]. In terms of height and weight, the UF phantom is between the two Iranian phantoms, but its higher BMI value indicates that the UF phantom is more obese than the other two phantoms. This issue was validated by comparing the amounts of soft tissue, adipose tissue and bone, which shield the internal organs of the UF and Iranian phantoms. This means that, although the main organs of the UF phantom in the abdomen–pelvis region are closer to the body surface, they are also more shielded by surrounding tissues located in beam path, especially by bone tissues, which have greater attenuation coefficients. It should be noted that an organ's position relative to that of bones affects the size of the dose absorbed by the organ. Because bones have a greater density than other tissues, if the amount of bone surrounding a particular organ increases, the dose delivered to this organ decreases. Thus, smaller doses to the internal organs of the UF phantom can be justified due to their greater protection by surrounding tissues. The results of this work support the Kalender statement, that for a given set of exposure parameters, the dose in the patient or phantom will vary greatly depending on the amount of tissues and fat layers around it, which attenuate the radiation intensity [24]. In addition, it is also asserted that there is a decrease in the dose absorbed by an organ by increasing the protective tissue layers, as shown by Karimi Shahri *et al.* [25]. Therefore, it seems that depth distributions of organs and their protection from external radiation are two competing factors; so that, for different organs, based on their shapes and locations, one of them will dominate the other.

As is well known, different types of body receive different amounts of radiation dose to the organs, but the degree of uncertainty in the amount of dose for non-reference subjects calculated from that of reference phantoms has not been quantified for the various percentiles as yet. Therefore, in this paper, we have presented the first results in the development of two Iranian 11-year-old non-reference phantoms and studies of the sizes of the absorbed dose. Considerable progress has been achieved.

Nevertheless, there is a limitation in this study, which should be taken care of in the future. Although these two phantoms were suitable for the purpose of this study, they were not standard for Iranian children. Hopefully, in the future, a library of Iranian standard phantoms will be developed, and this study will be extended to cover Iranian pediatric phantoms for a range of age groups. Thus, a library of phantoms will be incorporated into the database to help individualize the dose calculations for the Iranian pediatric population.
